# Prevention of Porcine Epidemic Diarrhea Virus With Nanotube‐Adjuvanted Oral DNA Vaccines

**DOI:** 10.1155/tbed/6727844

**Published:** 2026-01-11

**Authors:** Hsing-Chieh Wu, Thu-Dung Doan, Wan-Chen Chang, Min-Kung Hsu, Hsian-Yu Wang, Jiahorng Liaw, Chia-Jung Chang, Chun-Yen Chu, Nan-Hua Chen

**Affiliations:** ^1^ International Degree Program of Animal Vaccine Technology, International College, National Pingtung University of Science and Technology, Pingtung, 91201, Taiwan, npust.edu.tw; ^2^ Innovative Bioproducts Technical Service Center, National Pingtung University of Science and Technology, Pingtung, 91201, Taiwan, npust.edu.tw; ^3^ General Research Service Center, National Pingtung University of Science and Technology, Pingtung, 91201, Taiwan, npust.edu.tw; ^4^ Animal Biologics Production Center, National Pingtung University of Science and Technology, Pingtung, 91201, Taiwan, npust.edu.tw; ^5^ Graduate Institute of Animal Vaccine Technology, College of Veterinary Medicine, National Pingtung University of Science and Technology, Pingtung, 91201, Taiwan, npust.edu.tw; ^6^ College of Pharmacy, Taipei Medical University, 250 Wu Hsing Street, Taipei, 110, Taiwan, tmu.edu.tw; ^7^ R&D Division, Reber Genetics Co., Ltd., Taipei, Taiwan

**Keywords:** DNA vaccine, lactogenic immunity, nanotubes, oral, porcine epidemic diarrhea virus

## Abstract

Porcine epidemic diarrhea virus (PEDV) causes severe diarrhea in piglets. The ideal route of protection against PEDV for piglets is through passive (lactogenic) immunity, which is not provided by current inactivated and subunit vaccines on the market. In this study, we investigated whether a DNA vaccine encoding the full PEDV spike protein adjuvanted with cyclo‐peptide nanotubes (cPNTs) can provide protection against PEDV through active and passive immunity. For the active immunization experiment, piglets were vaccinated, and the immune response was analyzed, followed by a PEDV challenge test. In a separate experiment, to evaluate the passive (lactogenic) immunity elicited by the cPNTs‐adjuvanted DNA vaccine, pregnant sows in a local farm were immunized, and the survival of farrowed piglets was examined. The results showed that, in the active immunization experiment, the DNA vaccine elicited IFN‐γ and IL‐12 production in piglets. IgA antibodies were detected in the serum, and the expansion of CD4^+^ and CD8^+^ T cells was observed. Upon virus challenge, vaccinated piglets remained healthy, gained weight, and showed only mild signs of diarrhea, with minimal virus shedding (Ct value of 33, compared with 16 for the saline‐vaccinated control group). For the passive immunity experiment, results show that the DNA vaccine administered orally induced higher levels of IgA in the colostrum of vaccinated sows compared to mock vaccination. The survival rate of the farrowed piglets was higher at 84% for the DNA‐oral group compared to that of the mock vaccination group (68%). In conclusion, the cPNTs‐adjuvanted DNA vaccine can not only generate protective immunity through direct immunization of piglets but also induce lactogenic immunity in pregnant sows to protect farrowed piglets from PEDV infection.

## 1. Introduction

Porcine epidemic diarrhea virus (PEDV) infects pigs of all ages, especially newborn piglets between 1 and 3 weeks of age, resulting in morbidity and mortality rates up to 100%. After entering the host through the fecal–oral route, PEDV targets cells of the small intestine and leads to acute diarrhea, fever, vomiting, and anorexia [1, 2]. Most commercial vaccines available for PEDV control are traditional live attenuated or killed vaccines that offer limited protection [3] and often fail to induce lactogenic immunity in sows to protect their suckling piglets [4]. The development of more effective vaccines is needed.

Nucleic acid‐based vaccines represent a modern vaccine design approach that allows the direct expression of antigens in the host, mimicking actual viral infection. In this study, we constructed a plasmid DNA vaccine expressing the full PEDV spike protein. PEDV is an enveloped virus with a single‐stranded, positive‐sense RNA genome belonging to the Alphacoronavirus genus of the Coronaviridae family. The PEDV genome is ~28 kb long and consists of seven open reading frames (ORFs), including ORF1a, ORF1b, an accessory protein (ORF3), and four structural proteins: spike protein (*S*), envelope protein (*E*), membrane protein (*M*), and nucleocapsid protein (*N*) [5, 6]. The *S* protein resides on the viral surface and can be divided into two domains, *S*1 and *S*2. *S*1 contains most receptor binding sites, whereas *S*2 is mainly responsible for membrane fusion [7]. Most neutralizing antibodies are directed at the *S* protein, making it the most important antigen of PEDV for vaccine development [8–13].

The effective delivery of plasmid DNA across the cell membrane for protein expression poses a significant hurdle [14]. We will therefore employ a gene delivery vehicle, cyclo‐peptide nanotubes (cPNTs) [15, 16], as an adjuvant for improved delivery and vaccine efficacy [17–20]. Peptide rings can be synthesized from three repeating units of dipeptides, such as D‐Trp–Tyr (Figure 1C). The resulting rings can spontaneously self‐assemble through β‐sheet stacking to form nanotubes of various dimensions [21–23]. Since these cPNTs contain positively charged side chains such as tyrosine, they can bind to negatively charged DNA and facilitate its delivery across the lipid bilayer [24]. In vivo analysis demonstrated that cPNTs can persist in blood and exhibit low toxicity [25]. Importantly, cPNTs can facilitate the oral delivery of plasmid DNA through mucosal surfaces to the lung, spleen, liver, and reticuloendothelial system (RES) [15]. In our previous study in ducklings, we showed that a cPNTs‐adjuvanted DNA vaccine can elicit a significant humoral immune response [26]. The expression of the target antigen, VP2, was confirmed through immunohistochemical staining of the intestine and liver tissues from vaccinated ducklings. Analysis of antibody response revealed high levels of IgA antibodies in the serum and mucosal surfaces.

Figure 1DNA vaccine plasmid construction and illustration of the cPNT structure. (A) Map of the designed pTCY‐spike DNA plasmid. The PEDV *S* gene (4173 bp) was cloned and inserted into the multiple cloning site after the beta‐actin promoter. (B) Proper insertion of the *S* gene was confirmed by *Kpn*I restriction enzyme digestion. (C) Illustration of the structures of D‐Trp–Tyr and cyclo (D‐Trp–Tyr), which stack to form cPNTs.

**Figure:**
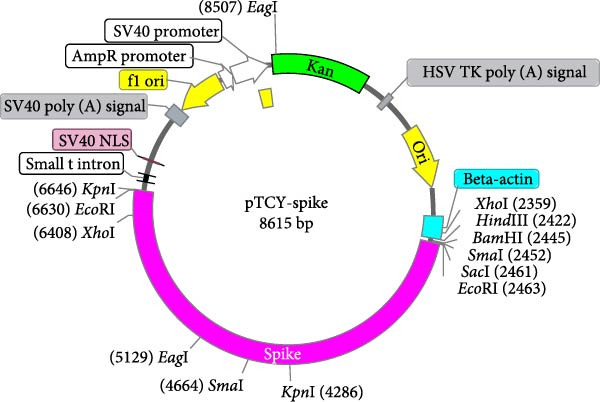
(A)

**Figure:**
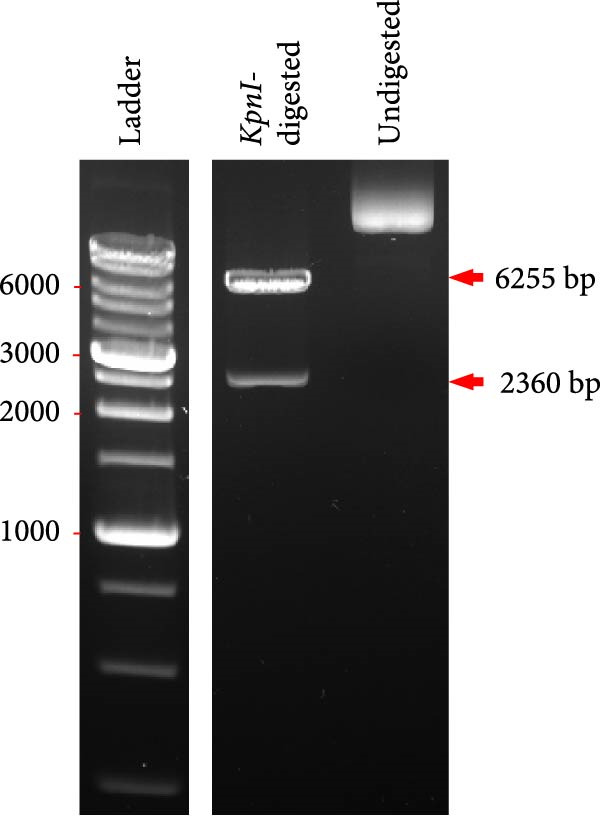
(B)

**Figure:**
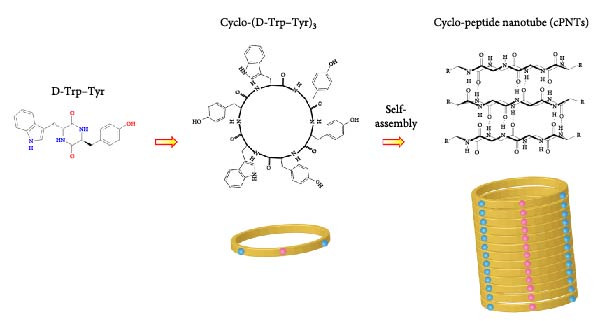
(C)

In this study, a DNA vaccine encoding the full PEDV S gene was adjuvanted with cPNTs. To evaluate the active immunity elicited by this vaccine, piglets were immunized and subsequently challenged with PEDV. In a separate experiment, to assess the passive (lactogenic) immunity elicited by the cPNTs‐adjuvanted DNA vaccine, sows in a local farm with potential exposure to PEDV were immunized, and survival of the farrowed piglets was evaluated.

## 2. Materials and Methods

### 2.1. Construction and Formulation of the pTCY‐spike DNA Vaccine With cPNTs

To construct a plasmid DNA vaccine expressing the full PEDV spike protein, the S gene from a virulent G2b strain, PT (GenBank Accession Number KP276252) [27], was synthesized (Synbio Technologies, Monmouth Junction, NJ, USA) for insertion into the pTCY expression vector [26] at the *Eco*RI site, and the sequence was verified by sequencing. In vitro spike protein expression has been verified (Figure S1). The resulting plasmid, named pTCY‐spike (Figure 1A), was propagated in *Escherichia coli* DH5α and digested with the restriction enzyme *Kpn*I to check insert orientation (Figure 1B). For vaccine use, the pTCY‐spike plasmid was purified using the EndoFree Plasmid Maxi Kit (Qiagen, Hilden, Germany) to ensure endotoxin levels <0.04 EU/μg, in line with FDA vaccine standards. The purified plasmids were diluted to the final concentration using sterile phosphate‐buffered saline (PBS) and stored at −20°C until use.

For cPNTs production, 5 mg of cyclo‐(D‐Trp–Tyr) (Bachem, Bubendorf, Switzerland) was dissolved in 15 mL of 50% alcohol in a glass beaker, sonicated for 5 min at room temperature, and incubated overnight at 37°C to evaporate the solvent [28]. The resulting dried powder of the cPNTs was scraped off and stored at 4°C.

To formulate the final DNA vaccine, 1.5 mg of cPNTs was added to 1 mL of solution containing 50 μg of pTCY‐spike plasmid, and the mixture was stirred overnight at 25°C to coat the plasmid with the cPNTs. The sterility of the vaccine was confirmed by the absence of growth on trypticase soy agar plates at 37°C (Difco, MD, USA), thioglycollate agar plates at 37°C (Difco), and Sabouraud dextrose agar plates at 25°C (Difco).

### 2.2. Vaccination of Piglets

**Figure 2:**
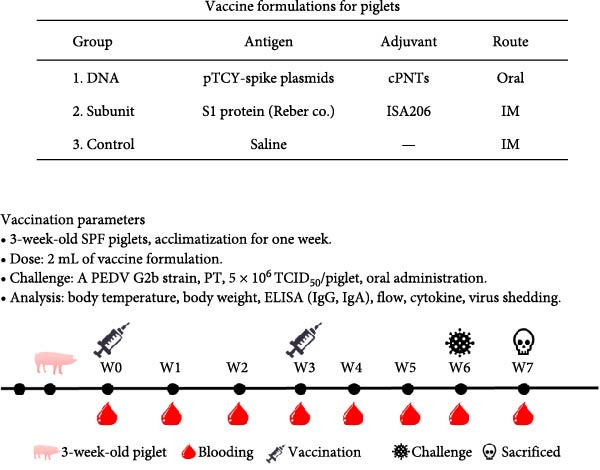
Vaccination formulations and vaccination schedule for piglets. The components of the vaccines used for the study are shown. A schedule for immunization and subsequent virus challenge is also presented.

Three formulations were prepared for vaccination: (1) DNA, (2) subunit, and (3) control (saline), at 2 mL per dose (Figure 2). The subunit vaccine is a commercial vaccine (Reber Genetics Co., Ltd., Taipei, Taiwan) of the *S*1 protein, adjuvanted with Montanide ISA206 W/O/W (Seppic SA, Paris, France). Nine healthy SPF piglets (purchased from the Agricultural Technology Research Institute, Miaoli, Taiwan) were introduced at 3 weeks of age, followed by a 1‐week acclimatization period. The piglets were randomly assigned to three groups for the three formulations. Vaccination was administered twice at 4 and 7 weeks of age (Figure 2).

Blood samples were collected after immunization for cytokine, T‐cell, and antibody analysis. The piglets were monitored daily for clinical symptoms. Body temperature (fever defined as rectal temperature above 39.4°C) and weight were recorded.

The SPF piglets were housed in an aseptic environment, with efforts made to minimize animal suffering. Animal experimental protocols (NPUST‐110‐083) were approved by the Animal Care and Use Committee at the National Pingtung University of Science and Technology, in accordance with the ethical rules and laws of NPUST.

### 2.3. Immune Analysis of Vaccinated Piglets: Cytokine Production

To determine cytokine levels after vaccination, blood samples were allowed to clot for at least 30 min before 10 min of centrifugation at 5000 rpm, and the resulting sera were collected. Protease inhibitors (Cat. Number 539134, Merck, Darmstadt, Germany) (1 μL per 100 μL of serum) were added to the serum samples before they were stored at −20°C. After all the serum samples were prepared, the levels of IFN‐γ, IL‐4, IL‐6, and IL‐12 in the samples were determined using the MILLPLEX Porcine Cytokine/Chemokine Magnetic Bead Panel (Merck KGaA, Darmstadt, Germany) following the manufacturer’s instructions.

### 2.4. Immune Analysis of Vaccinated Piglets: Cellular Immune Response

For the analysis of immune cells in vaccinated piglets, Ficoll‐Paque (GE Healthcare, St. Giles, Sweden) was added to whole blood samples to isolate peripheral blood mononuclear cells (PBMCs). After centrifuging the mixture at 525 × *g* for 30 min, the PBMC fraction was collected, washed, and resuspended in RPMI‐1640 (1 × 10^6^ cells/mL). For the analysis of the percentages of CD4^+^ and CD8^+^ T cells, mouse antiswine CD4^+^ and CD8^+^ antibodies (AbD Serotec, Oxford, UK) were applied to the PBMC samples in fluorescence‐activated cell sorting (FACS) binding buffer (1% BSA, 1% serum albumin, 0.01% sodium azide) for 30 min at 4°C. After washing, the cells were then incubated with PE‐labeled mouse anti‐pig CD4^+^ or FITC‐labeled anti‐pig CD8^+^ antibodies (BD Biosciences, Franklin Lakes, NJ, USA) at 4°C for 30 min in the dark. The percentages of CD4^+^ and CD8^+^ cells were determined by flow cytometry (BD Biosciences).

### 2.5. Immune Analysis of Vaccinated Piglets: IgG and IgA Antibody Production

To determine the serum IgG levels of the vaccinated piglets, the PEDV IgA Antibody Test Kit (IDEXX Laboratories, Westbrook, Maine, USA) was employed, substituting the secondary antibody conjugate with goat antiswine IgG‐HRP (1:5000) (KPL, Gaithersburg, MD, USA) for IgG detection. Serum samples were diluted 1:40 as instructed, and experimental procedures were carried out according to the manufacturer’s instructions. The absorbance was measured at 650 nm using the Anthos 2020 Microplate Reader (Anthos, Cambridge, UK). Results are calculated as *S*/*P* ratios, *S*/*P* =  (sample mean – negative control)/(positive control – negative control). *S*/*P* < 0.50 is defined as a negative result and *S*/*P* ≥ 0.50 as a positive result.

For IgA analysis, the same PEDV IgA Antibody Test Kit (IDEXX) was employed, using the original secondary antibody conjugate provided by the manufacturer.

### 2.6. Immune Analysis of Vaccinated Piglets: Serum Neutralizing Antibody

The PEDV‐neutralizing titer of serum from vaccinated piglets was determined. Briefly, test sera were heat‐inactivated at 56°C for 30 min and subsequently prepared to a two‐fold serial dilution in DMEM (Gibco, Grand Island, NY, USA) supplemented with tryptose phosphate broth (0.3%), yeast extract (0.02%), and 10 μg/mL of trypsin. The PT strain of PEDV (200 TCID_50_) was mixed with an equal volume of diluted sera and incubated at 37°C with 5% CO_2_ for 1 h. Following incubation, the mixture was added to a preformed monolayer of Vero cells in a 96‐well cell culture plate. After a 1‐h adsorption period at 37°C, the medium was removed, and the wells were washed twice with PBS. Subsequently, 100 µL of freshly supplemented DMEM was added to each well, and the plates were incubated at 37°C, 5% CO_2_ for 72 h. The serum neutralization (SN) titer was determined as the reciprocal of the highest serum dilution that completely inhibited cytopathic effects (CPEs).

### 2.7. PEDV Challenge Test of Vaccinated Piglets

Six weeks after the first vaccination, piglets were inoculated orally with 5 × 10^6^ TCID_50_/piglet of the PT virus (GenBank Accession Number KY929405). Clinical symptoms of the piglets were monitored daily, and clinical scores were assessed using the criteria shown in Table 1.

**Table 1:** Clinical symptom scoring criteria after PEDV challenge.

Clinical symptoms	Scoring criteria
Diarrhea	0: Fecal swab color and texture are normal. 1: Fecal swab color or texture abnormal. 2: Fecal swabs have abnormal color and texture.

Dehydration and weight loss	0: Weight loss within 0.5 kg compared with the previous day. 1: Lose 0.5–1 kg in weight compared to the previous day. 2: Weight loss of more than 1 kg compared to the previous day.

Anorexia	0: Normal eating. 1: Not much to eat. 2: Do not eat at all.

Vomit	0: No foreign matter around mouth. 1: Vomit remaining around mouth.

Depression	0: None. 1: Yes.

Rough hair	0: Smooth back hair. 1: Rough back hair.

Fecal swabs were taken daily after challenge for 7 days to determine virus shedding. Viral RNA extraction was performed with a PetNAD nucleic acid coprep kit (GeneReach Biotechnology Corporation, Taichung, Taiwan), followed by reverse transcription with the high‐capacity cDNA reverse transcription kit (Applied Biosystems, Waltham, MA, USA). Next, quantitative PCR was performed using the SYBR Green Supermix (Bio‐Rad, Hercules, CA, USA) on a CFX96 Touch Real‐Time PCR System (Bio‐Rad Laboratories Inc., Hercules, CA, USA), with primer pairs: F (5^′^‐CAC CTT GCA ATC TGT TA‐3^′^) and R (5^′^‐CCC TCA CCT TTA AAG CC‐3^′^). Each reaction had a total volume of 25 µL, containing 12.5 µL of 2x SYBR Green Supermix, 0.5 µL of each primer, 1 µL of DNA template, and 10.5 µL of nuclease‐free water. The thermal cycling protocol consisted of an initial denaturation at 95°C for 5 min, followed by 40 cycles of denaturation at 95°C for 15 s, annealing at 55°C for 30 s, and extension at 72°C for 45 s.

### 2.8. Vaccination of Sows

To study the passive immunity elicited by the DNA vaccine, three formulations were used for vaccination: (1) DNA‐oral, (2) DNA‐IM, and (3) Control. To produce the DNA‐oral vaccine, 1 mL of pTCY‐spike (260 μg/mL) was mixed with 1 mL of cPNTs (260 μg/mL) to produce 2 mL of vaccine for each dose for oral vaccination. For the DNA‐IM vaccine, 1 mL of pTCY‐spike (100 μg/mL) was mixed with 1 mL of CpG ODN (100 μg/mL) [29] to produce 2 mL of vaccine for each dose for intramuscular vaccination. CpG ODN has been shown to be an effective adjuvant by activating an immune response through Toll‐like receptor 9. The Control formulation contained saline. In a field trial (NPUST‐113‐064) at a local pig farm, 15 pregnant sows (Landrace Yorkshire crossbred, 1–2 years old, having farrowed 1–3 litters) were randomly selected and assigned to three groups corresponding to the three formulations. PEDV‐negative sows were primed 6 weeks before parturition with the vaccines and boosted 3 weeks prior to farrowing. During the vaccination period, the sows were monitored for adverse reactions to the formulations, and the number of abortions, if any, was recorded. At farrowing, the litter size and stillbirths were recorded. Throughout the experimental period (from priming to 12 days after farrowing), sows and the farrowed piglets were monitored for symptoms of diarrhea. Diarrhea samples were collected to confirm the presence of PEDV using RT‐PCR. Reserve transcription was performed as described earlier for RT‐qPCR. Then, PCR amplification (using the same set of primers as the RT‐qPCR described earlier) was carried out in a total reaction volume of 10 µL, comprising 5 µL of 2x KAPA Master Mix (Kapabiosystems, USA), 0.5 µL of each primer, 1 µL of DNA template, and 3 µL of nuclease‐free water. The thermal cycling conditions were as follows: initial denaturation at 95°C for 5 min; 35 cycles of denaturation at 95°C for 30 s, annealing at 55°C for 45 s, and extension at 72°C for 2 min; followed by a final extension at 72°C for 10 min. Amplification products were analyzed by electrophoresis on a 1% (w/v) agarose gel. Finally, the survival rate of farrowed piglets was calculated as survived piglets/total born for each experimental group.

All experimental protocols (NPUST‐110‐083, NPUST‐113‐064) for the animal trials were approved by the Animal Care and Use Committee at the National Pingtung University of Science and Technology. The experiments were conducted according to the ethical rules and laws of NPUST.

### 2.9. Immune Analysis of Vaccinated Sows: Antibody Analysis of Colostrum

Immune response of the vaccinated sows was studied by examining the levels of PEDV‐specific IgG and IgA antibodies in the colostrum. Samples of colostrum were collected at the earliest time possible after farrowing (within 24 h), centrifuged at 3000 rpm for 30 min. The whey layer was extracted and diluted 1:300 as instructed for antibody analysis using the same PEDV IgA Antibody Test Kit (IDEXX Laboratories) described in earlier sections. Finally, the PEDV neutralization titer of the colostrum was also evaluated.

### 2.10. Immune Analysis of Farrowed Piglets: Serum Neutralizing Antibody Against PEDV

The PEDV‐neutralizing titer of serum from farrowed piglets was determined. Serum samples were collected from three piglets for each sow, 3 days after parturition, for the virus neutralization test, which was performed as described in earlier sections.

### 2.11. Statistical Analysis

The general linear model in the statistical software SAS Version 9.0 (SAS Institute, Cary, NC, USA) was used for data analysis. Data points are expressed as the mean±standard deviation. Analysis of variance (ANOVA) and Duncan’s multiple comparison were performed to analyze the significance of the differences between groups. Significant differences (*p*‐value <0.05) are indicated by different letters.

## 3. Results

### 3.1. Plasmid DNA Containing the PEDV Spike Protein‐Encoding Gene Was Adjuvanted With cPNTs to Formulate a DNA Vaccine

The PEDV spike gene was successfully synthesized and inserted into the plasmid vector pTCY (Figure 1A,B). The resulting DNA plasmid (pTCY‐spike) was formulated with cPNTs (Figure 1C) as a vaccine. As a positive control, a commercial subunit vaccine was also included in the experiment, along with saline as the control group. SPF piglets were vaccinated twice with the three formulations for immune response evaluation and PEDV challenge tests (Figure 2).

### 3.2. Vaccination of Piglets With the DNA Vaccine Showed No Adverse Side Effects

The safety of the DNA vaccine was evaluated by monitoring the body temperature and weight changes of the vaccinated piglets. After primary immunization, the body temperature of the piglets in all three experimental groups remained below the normal body temperature threshold of 39.4°C (Figure 3A). In terms of weight gain, piglets in all groups showed comparable increases after primary immunization (Figure 3B). We conclude that the DNA vaccine meets the safety criteria for veterinary vaccines.

Figure 3Body temperature and weight of piglets after vaccination. (A) The body temperature of the immunized piglets was recorded for 1 week after vaccination, with 39.4°C as the fever threshold. (B) The body weights of the piglets were recorded 1 week after vaccination.

**Figure:**
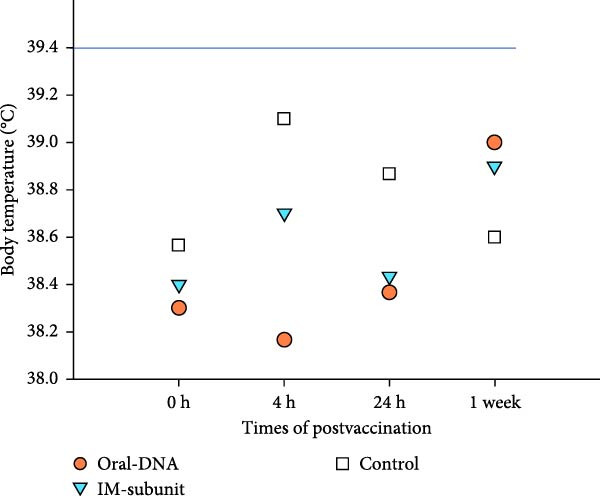
(A)

**Figure:**
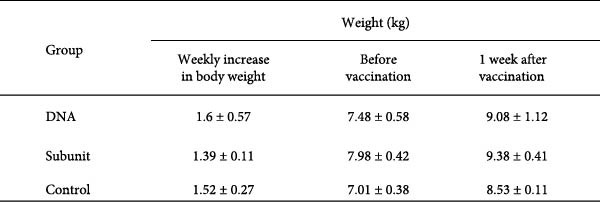
(B)

### 3.3. The DNA Vaccine Stimulated IFN‐γ and IL‐12 Production

To evaluate the immunogenicity of the DNA vaccine, the cytokine production profile of vaccinated piglets was evaluated. At 1–3 weeks after primary immunization, higher levels of IFN‐γ and IL‐12 were detected in the DNA vaccine group than in the control group (Figure 4A, B), demonstrating activation of the immune system and potentially a skewing toward a Th1‐type immune response. The production of IL‐4 and IL‐6 remained low at the background level (data not shown), indicating a lack of a Th2‐type immune response.

Figure 4Cytokine production and cellular immune response of immunized piglets. Serum samples from vaccinated piglets were collected, and the levels of IFN‐γ (A) and IL‐12 (B) were determined using ELISA. For cellular immune response analysis, PBMCs were also collected from the vaccinated piglets, and the percentages of CD4^+^ (C) and CD8^+^ (D) T cells were determined using flow cytometry. The data are presented as the means ± standard deviations. Different superscript letters indicate significant differences between the time points of the same vaccine formulation (*p* < 0.05).

**Figure:**
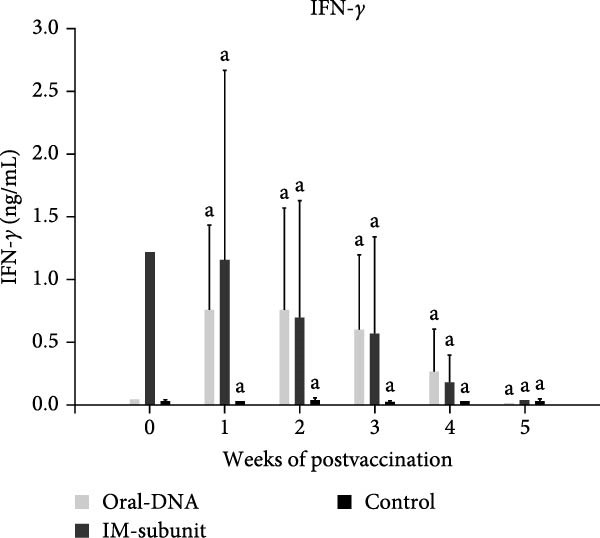
(A)

**Figure:**
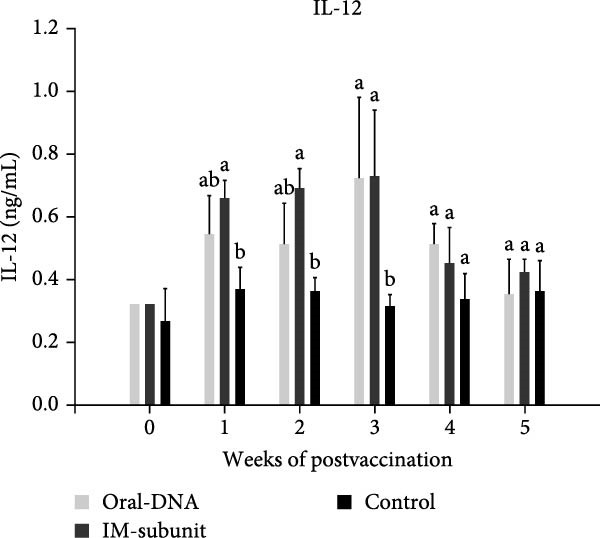
(B)

**Figure:**
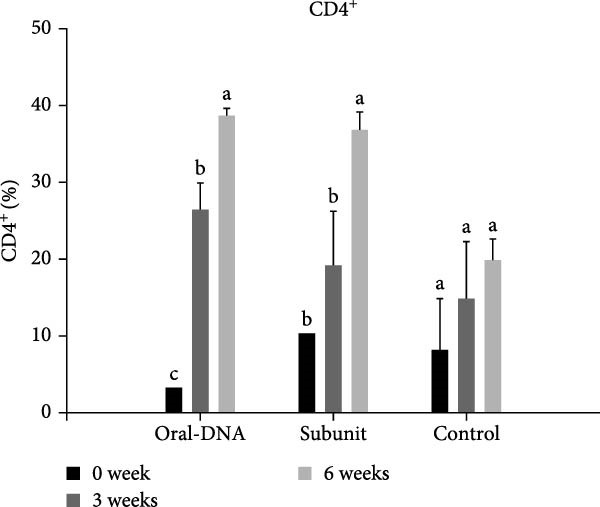
(C)

**Figure:**
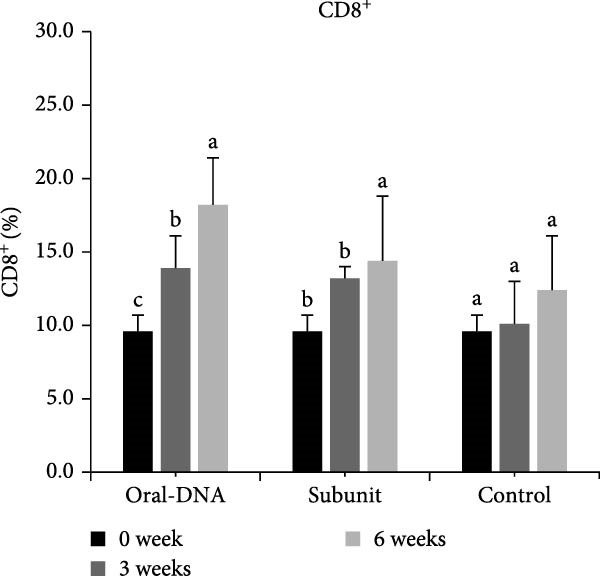
(D)

### 3.4. The DNA Vaccine Enhanced the Percentages of CD4^+^ and CD8^+^ T Cells in PBMCs

Cellular immunity induced by the DNA vaccine in piglets was studied. As shown in Figure 4C, the percentage of CD4^+^ T cells among PBMCs in the DNA vaccine group increased significantly from 3% at week 0%–38% at week 6 after primary immunization. Concomitantly, the percentage of CD8^+^ T cells increased from 8% to 17% (Figure 4D). This indicates that the DNA vaccine activated a cellular immune response.

### 3.5. The DNA Vaccine Elicited IgA and Neutralizing Antibody Production

The antibody response elicited by the DNA vaccine in piglets was studied. In terms of the systemic antibody response, the IgG level of the DNA vaccine group remained at the background levels even at week 6 after primary immunization (Figure 5A). In comparison, the commercial subunit vaccine induced IgG production 3 weeks after primary immunization. For mucosal antibody response elicited by the DNA vaccine, the IgA level was elevated 3 weeks after primary immunization (Figure 5B). Overall, the DNA vaccine generated an IgA antibody response, while the systemic IgG antibody response appeared low.

Figure 5Humoral immune response of vaccinated piglets. Serum samples were collected from the vaccinated piglets, and the levels of IgG (A) and IgA (B) were determined. Results are presented in *S*/*P* ratios, with values <0.50 defined as negative. Serum samples were also evaluated for PEDV neutralization titer (C), defined as the reciprocal of the highest serum dilution that completely inhibited cytopathic effects (CPEs). The data are presented as the means ± standard deviations. Different superscript letters indicate significant differences between the time points of the same formulation (*p* < 0.05).

**Figure:**
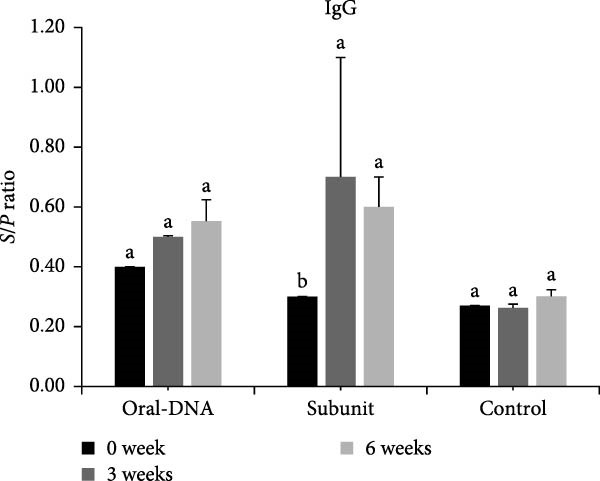
(A)

**Figure:**
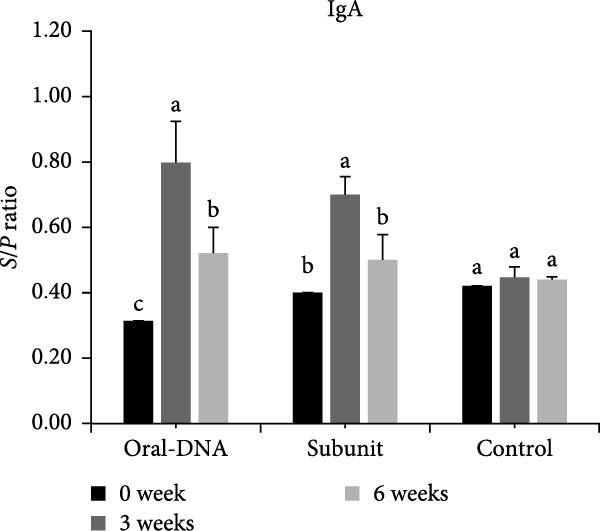
(B)

**Figure:**
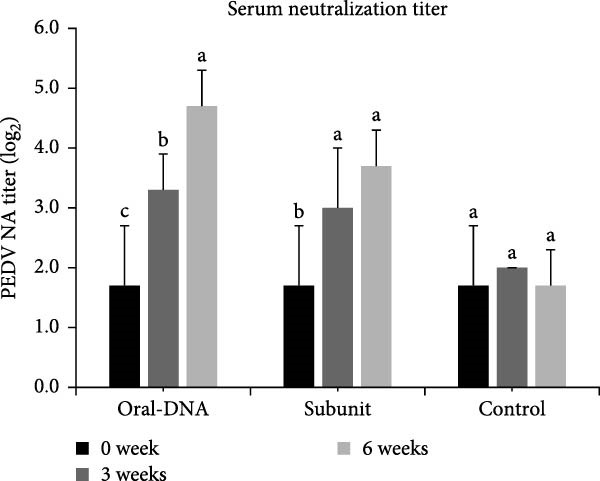
(C)

Analysis of serum neutralizing antibody showed that the DNA vaccine can induce neutralizing titers comparable to those of the subunit vaccine (Figure 5C).

### 3.6. The DNA Vaccine Protected Piglets Against PEDV Challenge

When vaccinated piglets were challenged with a virulent G2 strain of PEDV, those in the control group exhibited severe diarrhea, anorexia, dehydration, and rough hair, with a total clinical score of 52 (Table 2). In contrast, piglets vaccinated with the DNA vaccine showed only mild diarrhea, with a low clinical score of 7. The commercial subunit vaccine group also had a low clinical score of 3. A slight increase in body temperature can be seen for piglets in the DNA and control groups 2–3 days after challenge (Figure 6A). In terms of body weight gain, piglets in the control group gained only 0.8 kg 1 week after challenge, while those in the DNA vaccine group and subunit vaccine group gained up to 3.9 kg in the same period, clearly demonstrating the efficacy of the vaccines (Table 2). Analysis of viral shedding in fecal swabs revealed a low level of virus, with a Ct value of 33, in the DNA vaccine group (Figure 6B). In contrast, the control group showed a high level of virus, with a Ct value of 16. Overall, the piglets in the DNA vaccine group withstood the virus challenge test.

Figure 6Body temperature and virus shedding of vaccinated piglets after PEDV challenge. Vaccinated piglets were challenged orally with 5 × 10^6^ TCID_50_/piglet of the PT virus. (A) Body temperature was recorded for 7 days after the challenge. (B) Fecal swabs were collected 4 days after challenge, and quantitative RT‒PCR was performed to determine virus levels.

**Figure:**
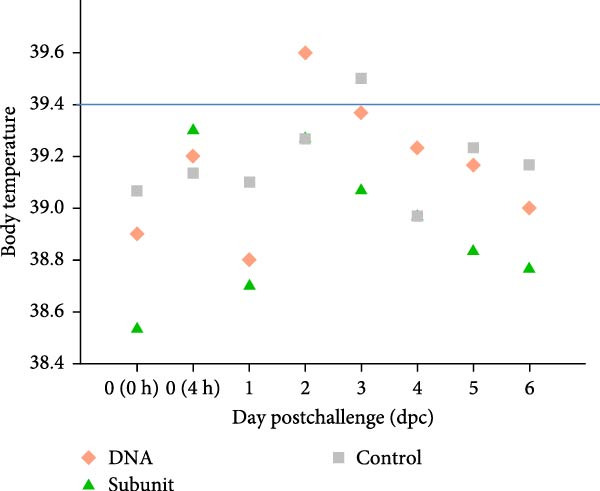
(A)

**Figure:**
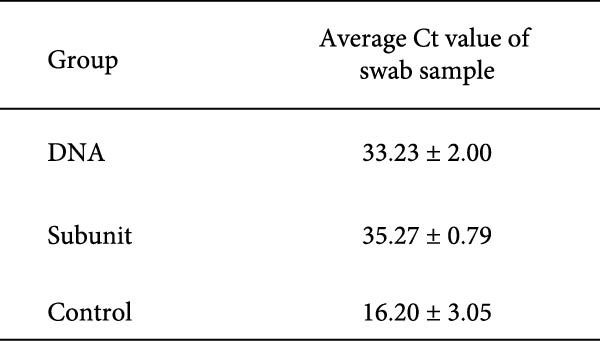
(B)

**Table 2:** Clinical scores and changes in the body weights of vaccinated piglets after PEDV challenge.

Group	Clinical symptoms					Body weight (kg)		
	Total^1^	Diarrhea	Anorexia	Dehydration and weight loss	Rough hair	Total gain/7 days	Before challenge	After challenge
DNA	7^b^	7	0	0	0	3.9^a^	25.6 ± 3.4	29.5 ± 3.1
Subunit	3^b^	2	1	0	0	3.7^a^	24.9 ± 4.6	28.5 ± 6.6
Control	52^a^	13	15	12	12	0.8^b^	22.4 ± 2.9	23.2 ± 2.7

*Note:* Different superscript letters indicate statistically significant differences between groups.

^1^Total score is the sum of the scores of the four categories of clinical symptoms from day 0 to day 6 postchallenge.

### 3.7. The DNA Vaccine Was Shown to be Safe for Pregnant Sows and Significantly Improved the Survival Rate of Farrowed Piglets

**Figure 7:**
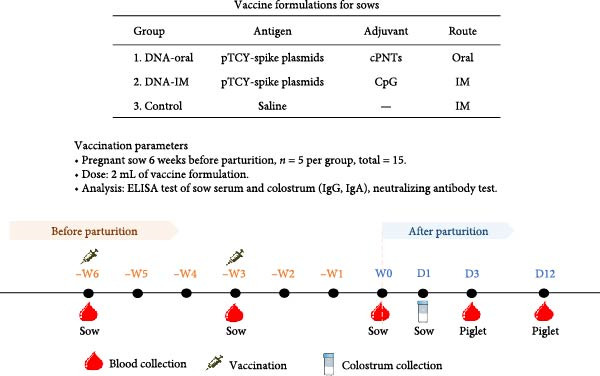
Vaccination formulations and vaccination schedule for sows. The components of the vaccines used for the study are shown. A schedule for immunization and subsequent immune analysis is also presented.

The ability of the DNA vaccine to induce passive immunity in pregnant sows to protect farrowed piglets was studied (Figure 7). The DNA vaccine was also formulated with the CpG adjuvant for intramuscular injection, since that is the commonly preferred method in the field. All three formulations for vaccination were shown to be safe for the sows, causing no adverse effects after inoculation. At delivery, sows in the DNA‐oral and DNA‐IM groups farrowed normally (Figure 8A). On the other hand, piglets from the control group were generally unhealthy: thin, some with suspected skin diseases (Figure 8B), vomiting (Figure 8C), and diarrhea for 8–10 days after birth (Figure 8D). Overall, the survival rate was significantly better for the vaccinated groups, 84% for the DNA‐oral group and 83% for the DNA‐IM group, compared to 68% in the control group (Figure 8E). This indicates that both the DNA‐oral and DNA‐IM vaccines elicited passive immunity for the farrowed piglets.

Figure 8Condition and survival rate of farrowed piglets. Pregnant sows were vaccinated, and the condition of farrowed piglets was recorded. Healthy piglets in the DNA‐oral vaccine group are shown (A). Piglets from the control group were generally unhealthy (B): thin, some with suspected skin diseases, vomiting (C), and diarrhea for 8–10 days after birth (D). Survival rates of farrowed piglets were calculated as survived piglets/total born for each experimental group (E).

**Figure:**
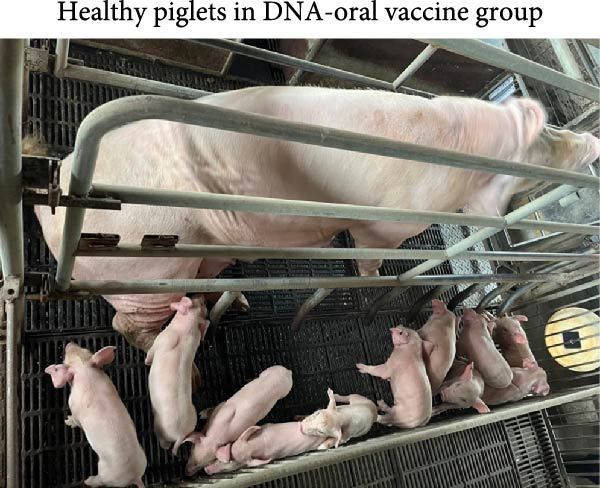
(A)

**Figure:**
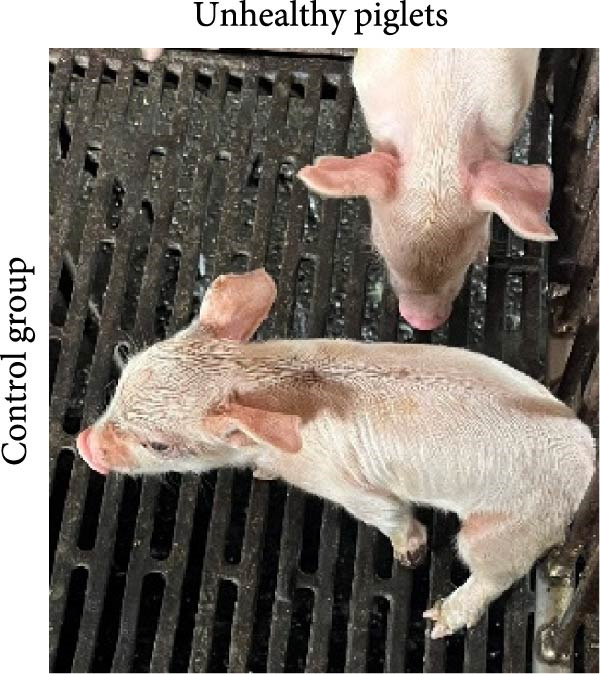
(B)

**Figure:**
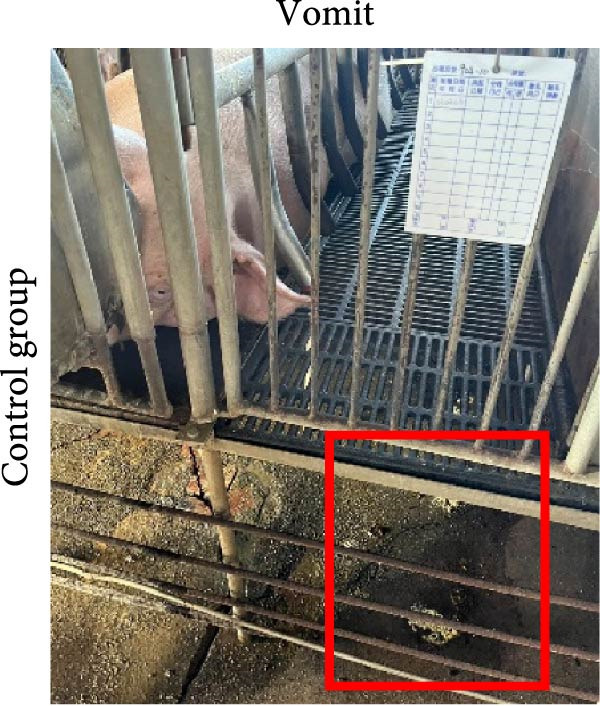
(C)

**Figure:**
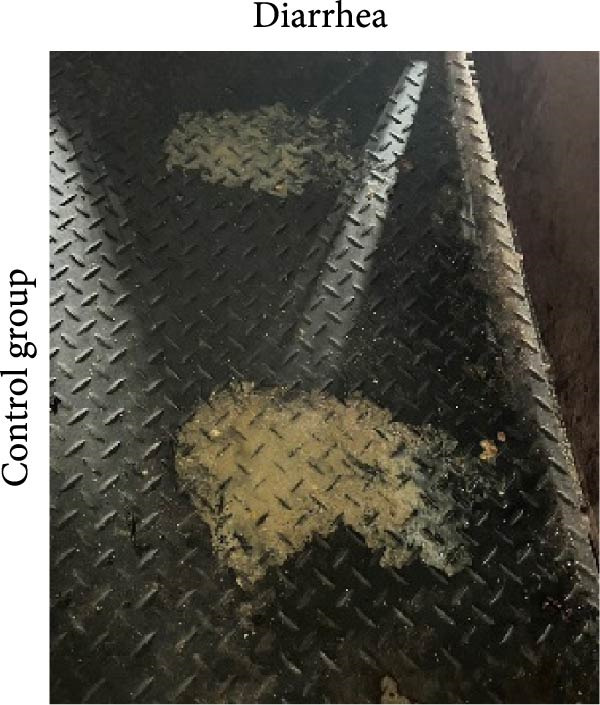
(D)

**Figure:**
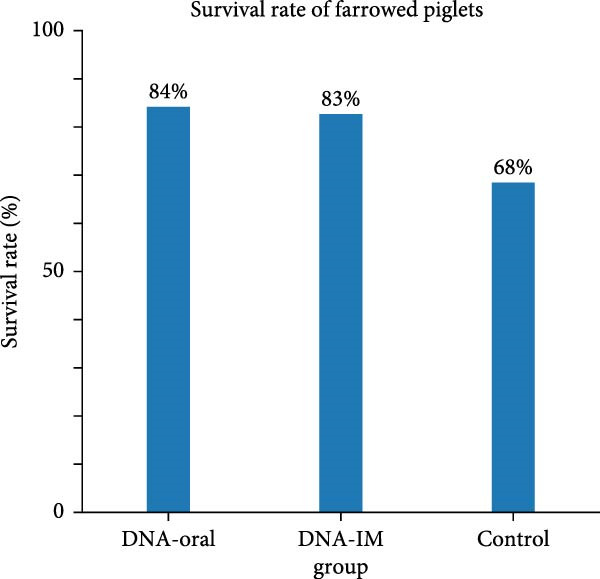
(E)

### 3.8. The DNA‐Oral Vaccine Elicited Higher Levels of IgA and Neutralizing Antibody in the Colostrum of Vaccinated Sows Than the DNA‐IM Vaccine

Analysis of IgG levels in the colostrum of vaccinated sows showed that the DNA‐IM vaccine elicited a higher level than the DNA‐oral vaccine (Figure 9A). In contrast, the DNA‐oral vaccine was more effective at eliciting IgA production than the DNA‐IM vaccine (Figure 9A). Finally, when the PEDV‐neutralizing titer was examined, data showed that the DNA‐oral vaccine elicited a higher titer than the DNA‐IM vaccine (Figure 9B).

Figure 9IgG, IgA, and neutralizing antibody levels in the colostrum of vaccinated sows. Colostrum samples were collected from the vaccinated sows, and the levels of IgG and IgA (A) were determined. Furthermore, colostrum samples were subjected to a virus neutralization test (B). The data are presented as the means ± standard deviations. Different superscript letters indicate significant differences between the time points of the same vaccine formulation (*p* < 0.05).

**Figure:**
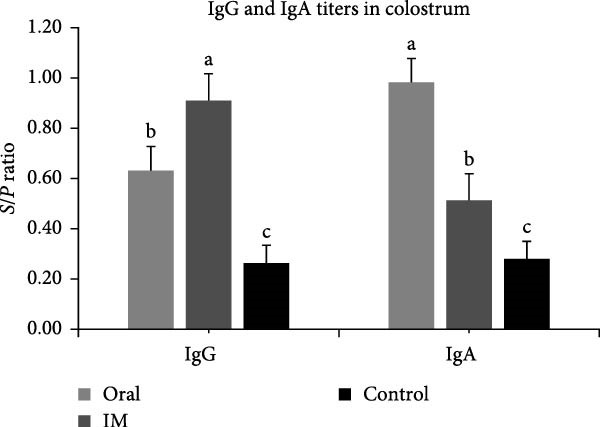
(A)

**Figure:**
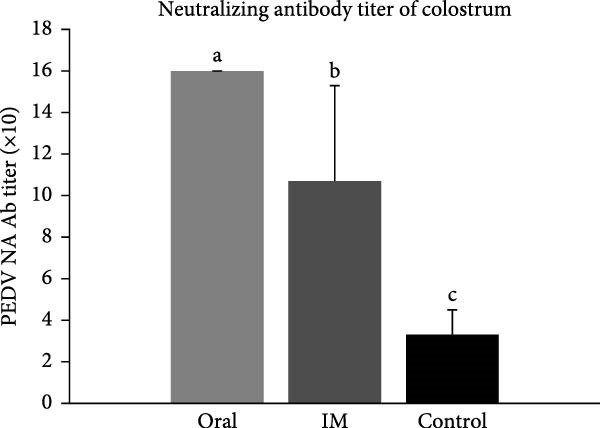
(B)

### 3.9. Farrowed Piglets From Vaccinated Sows Showed Elevated PEDV Neutralization Titers in Sera

**Figure 10:**
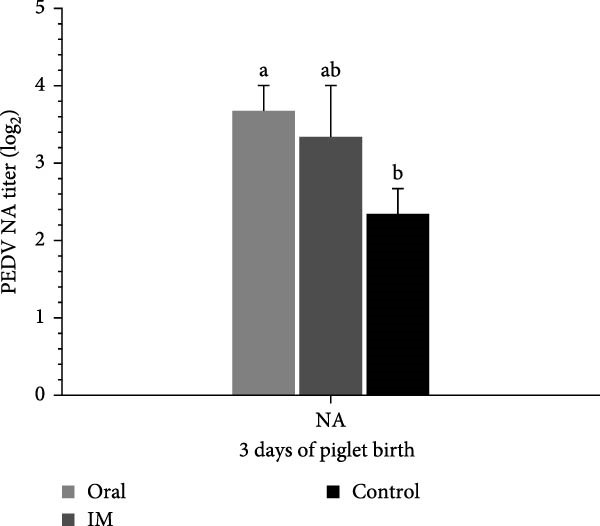
PEDV‐neutralizing titers in serum of farrowed piglets. Serum samples were collected from three piglets for each sow, 3 days after parturition, for the virus neutralization test. The serum neutralization (SN) titer was determined as the reciprocal of the highest serum dilution that completely inhibited cytopathic effects (CPEs). The data are presented as the means ± standard deviations. Different superscript letters indicate significant differences between the time points of the same vaccine formulation (*p* < 0.05).

The immunity conferred to the farrowed piglets against PEDV was further analyzed. Neutralization test of sera from the farrowed piglets showed that the vaccinated groups (DNA‐oral and DNA‐IM) had higher titers of antibodies against PEDV than the control group (Figure 10), indicating successful transfer of neutralizing antibodies from the sows to the piglets.

## 4. Discussion

Our formulation of an orally administered DNA vaccine adjuvanted with cPNTs represents a novel solution for the vaccination against PEDV. When used to vaccinate piglets directly, the cPNTs‐adjuvanted DNA vaccine elicited cellular immunity as well as IgA antibody production. A challenge test confirmed the protective efficacy of the DNA vaccine. Furthermore, the DNA vaccine also induced lactogenic immunity in pregnant sows to protect farrowed piglets from PEDV in the field. When successfully developed, this DNA vaccine, thermal‐stable and less costly to produce than subunit vaccines, could offer distinct advantages on the market.

In the direct vaccination experiment of piglets, the immune response evoked by the DNA vaccine appeared to be more Th1‐focused (cellular immunity), a hallmark of successful nucleic acid‐based vaccines. In addition to the Th1‐type cytokines induced, CD4^+^ and CD8^+^ T cells were observed to expand significantly after vaccination, providing evidence for the induction of cellular immunity. In terms of humoral immunity, the low level of systemic IgG antibody detected is particularly notable. Low IgG levels indicate a weak humoral immune response. However, IgA could still be detected, which may be even more important than IgG since it represents mucosal immunity. The presence of IgA on mucosal surfaces such as the gut could aid in virus clearing. This phenomenon of IgA production concurrent with low IgG production was observed in our previous study using a DNA vaccine adjuvanted with cPNTs against goose parvovirus [26], corroborating the immune response particular to this vaccine design and vaccination schedule.

Among the commercially available PEDV vaccines and those in development, the protective profile can be separated into two major categories: (1) inactivated or subunit vaccines administered intramuscularly that elicit a systemic IgG response [30] and (2) attenuated vaccines administered orally that can generate mucosal immunity, possibly through local activation of the gut‐associated mucosal system [31]. Inactivated and subunit PEDV vaccines are preferred by pig veterinarians and producers because of safety issues; however, the protection afforded is often limited [30]. On the other hand, live PEDV vaccines can generate a protective immune response and mucosal immunity [32]. However, as shown in previous studies, virus shedding from live vaccines is a real concern [31]. The development of nucleic acid vaccines can potentially address the major shortcomings of the two categories of vaccines by allowing antigen expression at mucosal sites without the use of a live agent.

Since PEDV mainly affects young piglets, the ideal prevention strategy against PEDV infection would be the vaccination of sows to confer lactogenic immunity [33]. Our field trial confirmed that the cPNTs‐adjuvanted DNA vaccines can indeed induce passive immunity to protect farrowed piglets. Furthermore, the vaccination of sows using two different vaccination routes, oral versus intramuscular, produced two sets of contrasting immune responses, with the oral (mucosal) vaccination method leading to more IgA antibody in the sow colostrum. A higher level of IgA antibody may indicate better mucosal immunity and thus better protection against pathogens on mucosal surfaces [34]. Nevertheless, similar survival rates were observed for both DNA‐oral and DNA‐IM groups, indicating that the DNA vaccine may also be developed for the intramuscular route of immunization, the currently preferred method in practice.

## 5. Conclusions

We have shown that the cPNTs‐adjuvanted DNA vaccine can not only generate protective immunity through direct immunization of piglets but also induce lactogenic immunity in pregnant sows to protect farrowed sows from PEDV infection.

## Conflicts of Interest

The authors declare no conflicts of interest.

## Author Contributions

Hsing‐Chieh Wu, Thu‐Dung Doan, and Wan‐Chen Chang performed the experiments and analyzed the data. Min‐Kung Hsu performed the gene analysis and design. Hsian‐Yu Wang and Jiahorng Liaw produced the nanotubes. Chia‐Jung Chang prepared the subunit vaccine and the PT virus. Chun‐Yen Chu conceived the study and wrote the manuscript. Hsing‐Chieh Wu and Thu‐Dung Doan contributed equally to this work and should be listed as co‐first authors.

## Funding

This work was supported by the National Science and Technology Council, Taiwan, R.O.C. (Grants MOST 110‐2622‐8‐020‐004, NSTC 111‐2622‐8‐020‐003, and NSTC 112‐2622‐8‐020‐001).

## Supporting Information

Additional supporting information can be found online in the Supporting Information section.

## Supporting information


**Supporting Information** Expression of pTCY‐spike DNA in BHK‐21 cells was confirmed by Western blotting. BHK‐21 cells were transfected with the pTCY‐spike DNA construct, and cell lysates were collected at 24 and 48 h post‐transfection for Western blot analysis.

## Data Availability

The data that support the findings of this study are available from the corresponding author upon reasonable request.
