# Supplementary material for: Prevention of Porcine Epidemic Diarrhea Virus With Nanotube‐Adjuvanted Oral DNA Vaccines
Source: Transbound Emerg Dis. 2026 Jan 11;2026:6727844. doi: 10.1155/tbed/6727844 (PMC12791578; doi:10.1155/tbed/6727844)
Supplement: Supplementary file 1 — Supporting Information Expression of pTCY‐spike DNA in BHK‐21 cells was confirmed by Western blotting. BHK‐21 cells were transfected with the pTCY‐spike DNA construct, and cell lysates were collected at 24 and 48 h post‐transfection for Western blot analysis. [file TBED-2026-6727844-s001.pptx]

## Slide 1
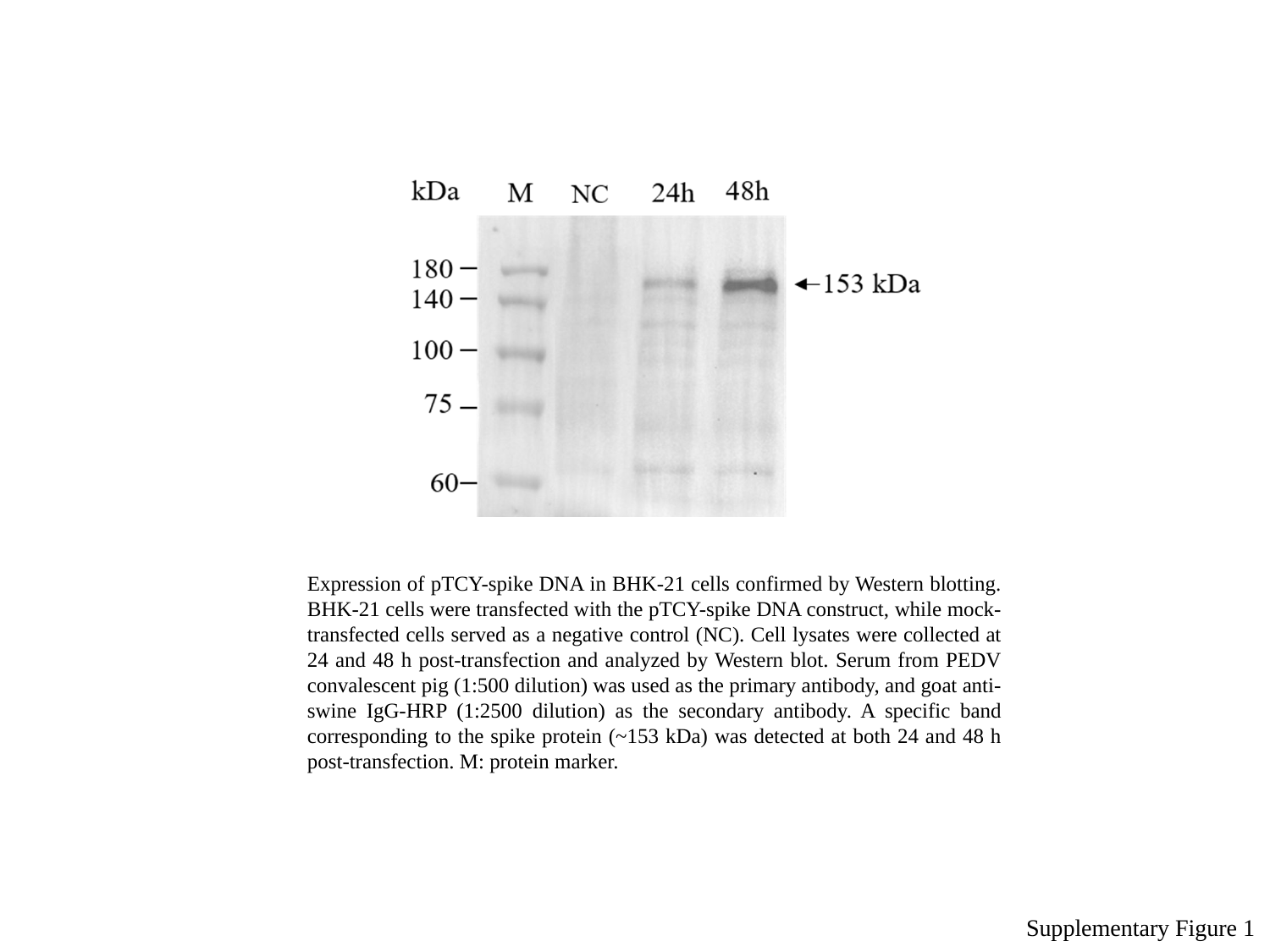

Expression of pTCY-spike DNA in BHK-21 cells confirmed by Western blotting. BHK-21 cells were transfected with the pTCY-spike DNA construct, while mock-transfected cells served as a negative control (NC). Cell lysates were collected at 24 and 48 h post-transfection and analyzed by Western blot. Serum from PEDV convalescent pig (1:500 dilution) was used as the primary antibody, and goat anti-swine IgG-HRP (1:2500 dilution) as the secondary antibody. A specific band corresponding to the spike protein (~153 kDa) was detected at both 24 and 48 h post-transfection. M: protein marker.
Supplementary Figure 1
